# Knowledge, Attitudes, and Stigma: The Perceptions of Tuberculosis in Equatorial Guinea

**DOI:** 10.3390/ijerph19148227

**Published:** 2022-07-06

**Authors:** Marta Vericat-Ferrer, Alba Ayala, Policarpo Ncogo, Juan Eyene-Acuresila, Belén García, Agustín Benito, María Romay-Barja

**Affiliations:** 1 Máster en Epidemiología, Dpto. Medicina Preventiva y S.P. y Microbiología, Universidad Autónoma de Madrid, 28029 Madrid, Spain; martuchicatfe@gmail.com; 2 Centro Nacional de Medicina Tropical, Instituto de Salud Carlos III, 28029 Madrid, Spain; abenito@isciii.es (A.B.); mromay@isciii.es (M.R.-B.); 3 Fundación Estatal, Salud, Infancia y Bienestar Social (FCSAI), 28029 Madrid, Spain; pncogo@psglobal.es (P.N.); bgarcia@fcsai.es (B.G.); 4 Ministerio de Salud y Bienestar Social, Malabo, Equatorial Guinea; pastorredimido@gmail.com; 5 Centro de Investigación Biomédica en Red de Enfermedades Infecciosas (CIBERINFEC), 28029 Madrid, Spain

**Keywords:** tuberculosis, knowledge, attitudes, stigma, Equatorial Guinea

## Abstract

Tuberculosis remains one of the major causes of morbidity and mortality in Equatorial Guinea, with an estimated incidence of 280 per 100,000 inhabitants, an estimated mortality rate of 96 per 100,000 inhabitants, and a treatment non-adherence rate of 21.4%. This study aimed to identify the factors associated to TB-related knowledge, attitudes, and stigma in order to design community intervention strategies that could improve TB diagnostic and treatment adherence in Equatorial Guinea. A nationwide cross-sectional survey of 770 household caregivers was conducted in Equatorial Guinea about TB knowledge, attitudes, and practices. Knowledge, attitude, and stigma scores were calculated through correct answers and the median was used as cut-off. Associated factors were analyzed calculating prevalence ratio (PR) and a 95% confidence interval (95% CI) through Poisson regression with robust variance. The percentage of women was 53.0% and median age was 46 years (IQR: 33–60). The percentage of caregivers with high TB related knowledge was 34.9%, with a bad attitude (52.5%) and low stigma (40.4%). A greater probability of having good knowledge was observed in those 45 years old or less (PR: 1.3, 95% CI: 1.1–1.6), those with higher education level (PR: 1.4, 95% CI: 1.1–1.8) and higher wealth (PR: 1.4, 95% CI: 1.0–2.0), while sex (PR = 0.8, 95% CI: 0.6–0.9), religion (PR = 1.4, 95% CI: 1.0–1.8), and good knowledge (PR = 1.4, 95% CI: 1.2–1.7) were associated with good attitudes. Wage employment (PR = 95% CI: 1.2–1.4), feeling well informed (PR = 0.7, 95% CI: 0.6–0.8), having good TB knowledge (PR = 1.3, 95% CI: 1.1–1.7), and some sources of information were associated with having lower TB-related stigma. This study found that a high percentage of caregivers in Equatorial Guinea lack important knowledge about TB disease and have bad attitudes and high TB-related stigma. Given the epidemiological situation of TB in the country, it is urgent to improve TB knowledge and awareness among Equatorial Guinea’s general population.

## 1. Introduction

Tuberculosis (TB) is the most common cause of death from a single infectious pathogen [1], with 29% of the nine million TB cases occurring in sub-Saharan Africa [2]. The cumulative reduction of TB incidence between 2015 and 2020 was 11%, far from the End TB Strategy milestone adopted by all WHO Members States in 2014 [3].

Human TB is a communicable disease caused by bacteria known as the Mycobacterium tuberculosis complex that mainly affects the lungs, making pulmonary disease the most common presentation. The main mode of transmission is through the inhalation of airborne infective aerosols. Tuberculosis is a preventable and curable disease, but its long treatment, lasting 6 to 12 months, makes its adherence difficult.

TB remains one of the major causes of morbidity and mortality in Equatorial Guinea, with an estimated incidence rate of 280 per 100,000 inhabitants in 2020 [4] and an estimated mortality rate of 96 per 100,000 inhabitants in 2021, with a TB case fatality ratio of 36% [4]. The TB effective treatment coverage is 34% [5]. In 2019, the estimated proportion of multidrug resistant TB (resistant to rifampicin and isoniazid) and rifampicin resistant TB was 2.5% of new cases and 37% of previously treated cases [6]. The estimated non-adherence to treatment rate in TB patients in Equatorial Guinea is 21.4% [7].

The National Tuberculosis and Leprosy Control Program (PNLP) in Equatorial Guinea is seeking to expand access to TB diagnosis and treatment. Care-seeking behavior and adherence to TB treatment tends to improve when the population has adequate knowledge about TB [8,9]. More information on the population’s knowledge, attitudes, and practices (KAP) about tuberculosis needs to be gathered in order to design public health interventions [10]. WHO has designed and validated a KAP survey for tuberculosis that can identify gaps in TB knowledge, attitudes, stigma, and behavioral patterns that may make TB control more difficult. It can also identify some factors that might be influencing these gaps and specify the best sources of information to improve TB prevention and control.

Adequate community knowledge about TB is crucial to guarantee that diagnosis, treatment, and preventative measures are correctly applied. However, misconceptions about TB frequently persist among communities [11]. TB disease can cause economic vulnerability, marginalization, stigma, and discrimination [12,13]. Poor knowledge and attitudes within the community can lead to a TB patient being stigmatized and often feeling shame and guilt. Poor knowledge, bad attitudes, and stigmatization could affect both the delay in diagnosis and treatment adherence [14].

Factors usually associated with TB knowledge, attitudes, and stigma are sex, age, and area of residence [11,15,16,17] together with education, employment, and wealth [11,15,16,17,18]. Bad attitudes and stigma are also frequently associated with poor disease knowledge [19]. Rural and urban populations differ in their cultural practices, socioeconomic and demographic characteristics, availability, and accessibility to health services. Urban populations are generally younger and better educated than rural populations [20]. Rural areas are linked with increased levels of poverty together with diminished access to healthcare facilities [11]. Both rural and urban populations should be taken into account to improve the efficacy and efficiency of TB control interventions.

Despite the high TB incidence, this is the first study that aimed to assess TB community knowledge, attitudes, and stigma in Equatorial Guinea and associated factors. This study may be helpful to develop appropriate educational and communication strategies aimed at improving disease control.

## 2. Materials and Methods

### 2.1. Study Area and Population

Equatorial Guinea is located in the Gulf of Guinea, with a surface area of 28,051 km^2^, a total estimated population of 1,225,377 inhabitants and 24% of rural population [21]. Equatorial Guinea consists of two regions: a mainland region and an insular region. Bioko is the largest island and home to the country’s capital, Malabo, while 72% of the nation’s population lives in the mainland region. The rural population has a lower level of education than the urban population (university studies 2% vs. 10%, respectively) [21].

### 2.2. Study Design, Sample, and Data Collection

**Figure 1:**
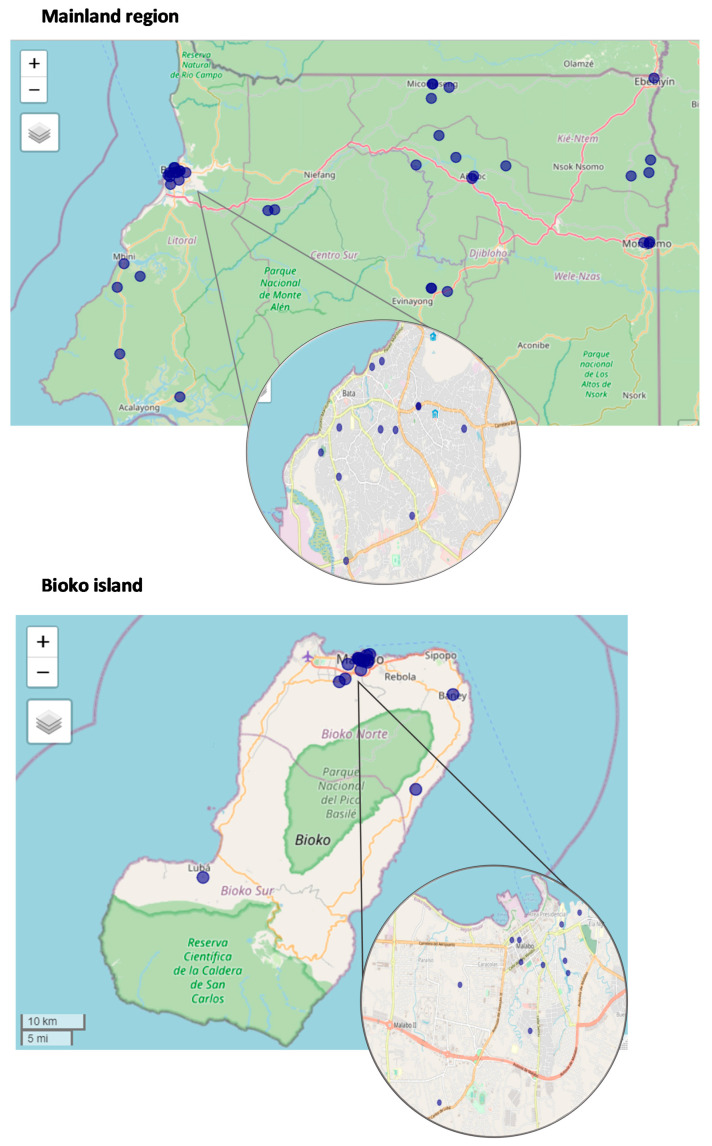
Communities of Equatorial Guinea where the interviews were carried out.

A nationwide cross-sectional survey was designed to determine tuberculosis-related knowledge, attitudes, and practices in Equatorial Guinea rural and urban household caregivers aged 18 years and older. Data were collected in October 2020 through a KAP survey in 55 communities of the mainland region and Bioko Island (Figure 1). The sample size was calculated using a 95% confidence interval, 5% error margin, and an expected proportion of good knowledge of 50%. A design effect of 2 was considered for a complex sample [22]. A multistage cluster random sampling was implemented. First, 14 rural villages and 41 urban neighborhoods were randomly selected with probability proportional to size to better assure representativeness in the sample design. Second, households were randomly selected from an updated census from each cluster provided by the head of the village or neighborhood. Only three households refused to participate in the survey. A total of 770 individuals were included in the study. The questionnaire, based on the validated WHO TB knowledge, attitude, and practice survey [23], was previously tested and translated into the main local languages, Fang and Spanish.

### 2.3. Variables

#### 2.3.1. Knowledge Score

A knowledge score [15,16] was calculated by adding values of 13 correct questions about knowledge and beliefs of TB symptoms, risk perception, transmission and prevention mechanisms, and treatment. The answers considered as having good knowledge were: TB is a serious disease; TB is caused by bacteria or germs; coughing, coughing with blood and chest pain are the symptoms of TB; a person can get TB through the air when a person with TB coughs or sneezes; TB can be prevented; TB can be cured; covering your mouth and nose when coughing or sneezing prevents TB; everybody can be infected with TB; TB is treated with specific medical drugs; the treatment is free of charge (Tables S1 and S2). This scale ranged from 0 to 13, with higher values indicating greater TB-related knowledge. The score was then categorized into high and low knowledge using the median of correct answers as cut-off point.

#### 2.3.2. Attitudes Score

Attitudes scores were created to assess the factors associated with caregivers’ attitudes towards tuberculosis [19]. The following answers were considered a good attitude (Table S3): said that they could get TB; mentioned “I would cope with it” and “surprise” as feeling if they were diagnosed with TB; said that they would talk about their TB diagnoses with a doctor/health worker and others; and said that they would go to a health facility if they had TB symptoms. The answers were scored with one point and the final attitude score was computed out of 7. The median was used as a cut off: those with a low score or equal to the median were considered as having a bad attitude, and a high score as having a good attitude.

#### 2.3.3. Stigma Score

The answers considered as low stigma towards people with TB were (Table S4): “Which statement is closest to your feeling about people with TB?—feel compassion and a desire to help to people with TB”; “In your community, how is a person who has TB usually treated?—The community mostly supports and helps him or her; and do not feel shame if they were diagnosed with TB”. The answers were scored with one point and the final stigma score was computed out of 3. The median was used as a cut-off: those with a low score or equal to the median will have a high level of stigma, while those with a high score will have a low level of stigma.

#### 2.3.4. Socio-Economic Factors

Age, sex, marital status, education, employment, and area of residence were included as socio-economic factors. A socio-economic index was constructed through principal component analysis using variables about quality housing, household-owned assets, water and electricity access, and sanitation conditions [24,25]. The measure obtained by the first principal component was separated into quintiles to identify the household wealth status. An overcrowding index was calculated with the number of cohabitants divided by the number of rooms, excluding the kitchen. This index was separated into two categories by the median.

### 2.4. Data Analysis

A descriptive analysis of participants’ characteristics and their TB knowledge was carried out using frequency tables. The differences by area in socio-demographic characteristics and TB knowledge were evaluated with a chi-square test for independence. In order to identify the socioeconomic factors associated with high knowledge, good attitudes, and low stigma, multiple regression models were carried out. Poisson regressions with robust variance were performed to calculate prevalence ratios (PR) and 95% confidence intervals (95% CI), in order to avoid odds ratio overestimations for prevalence rates above 10% [26]. Forward and backward stepwise procedures were applied for the model selection. Finally, forward selection was chosen because it presented fewer variables in the model and lower Akaike Information Criterion (AIC) and Bayesian Information Criterion (BIC) than the backward method. Stata 17.0 software was used for the data analysis.

## 3. Results

A total of 770 household caregivers were interviewed about their TB knowledge, beliefs, and attitudes, 560 (72,7%) of whom lived in urban area. Significant differences by place of area were found in socio-economic variables (Table 1). Caregivers were younger in urban areas (mean age = 51.5 years) than in rural areas (45.1 years). In rural areas, caregivers had a lower educational status (46.7% of rural vs. 29.8% urban of people with primary and lower education, *p* < 0.001), less wage-employment (19.0% vs. 32.7%, *p* < 0.001), are poorest (52.4% vs. 7.9%, *p* < 0.001), and there was less overcrowding (66.7% vs. 43.8%, <0.001). The presence of a TB case was also less frequent in rural families (31.4% vs. 39.0% *p* = 0.057).

Most of the household caregivers said that TB is a serious disease (93.2%), but only 32.5% thought that there was TB in their community (Table S1), found more frequently in the rural population (47.6%, *p* < 0.001). More than half of the respondents did not know what causes TB (64.6%) and answered that TB is transmitted by sharing dishes/glasses (63.5%). The most frequently mentioned TB signs and symptoms were coughing (65.5%) and weight loss (33.2%).

Less than half of the caregivers (44.0%) said that everybody could be infected with TB (Table S2), while other most frequent answers were “only alcoholics” (16.5%) and “only drug users” (11.0%). Most of the caregivers (78.4%) knew that TB can be prevented, but the most frequent preventive measure mentioned was to avoid sharing dishes, glasses, and cutlery (52.2%), while 30.4% responded “covering mouth and nose”. Most caregivers said that TB can be cured (91.7%) with “specific drugs” (91.7%), that they should go to hospital for TB treatment (98%), and 74.2% knew that treatment is free of charge (mainly in rural areas *p* = 0.040). The median of TB knowledge score was 8 (range: 1–13) and only 34.9% of respondents had a score above the median.

The most frequent emotions expressed about TB attitudes when asked about how they would feel if they were diagnosed with TB were fear (34.4%), sadness, or hopelessness (32.3%), and only 10.7% said that they could cope with it (Table S3). When asked who they would talk to if they were diagnosed with TB, the most frequent answers were a doctor or another medical worker (46.3%). All the caregivers who mentioned the doctor/health worker also mentioned family members (43.2%) or their partner (41.8%). In the case of TB health care-seeking behavior, almost all of the respondents said they would go to the hospital if they thought they had TB symptoms (93.0%) and only 0.4% of participants said that they would go to a traditional healer. The median of the TB attitude score was 2 (range: 0–7) and almost half of the caregivers had a good attitude towards TB disease (47.5%).

Regarding stigma (Table S4), asked about their feelings towards people with TB, most of the caregivers said that they feel compassion and a desire to help (74.3%), while 17.7% said that they feel compassion but that they tend to stay away from these people. Asked about how their community treats people with TB, almost half of the respondents considered that the community mostly supports and helps them (48.2%). The median of the TB-related stigma score was 2 (range: 0–3) and only 40.4% of the respondents had a low TB-related stigma score.

**Figure 2:**
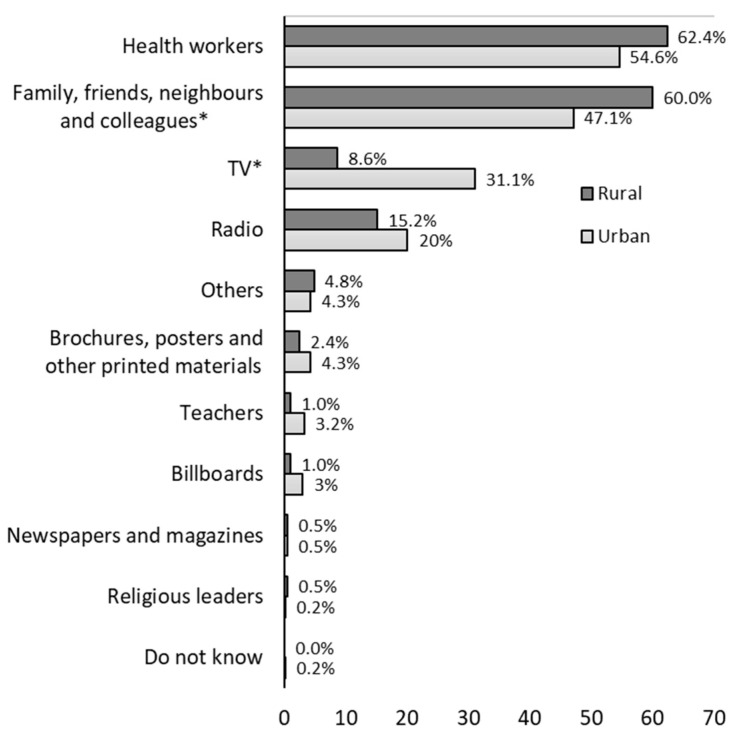
Where did you first hear about tuberculosis or TB? * Significant chi-square test of differences by area at 5% level.

**Figure 3:**
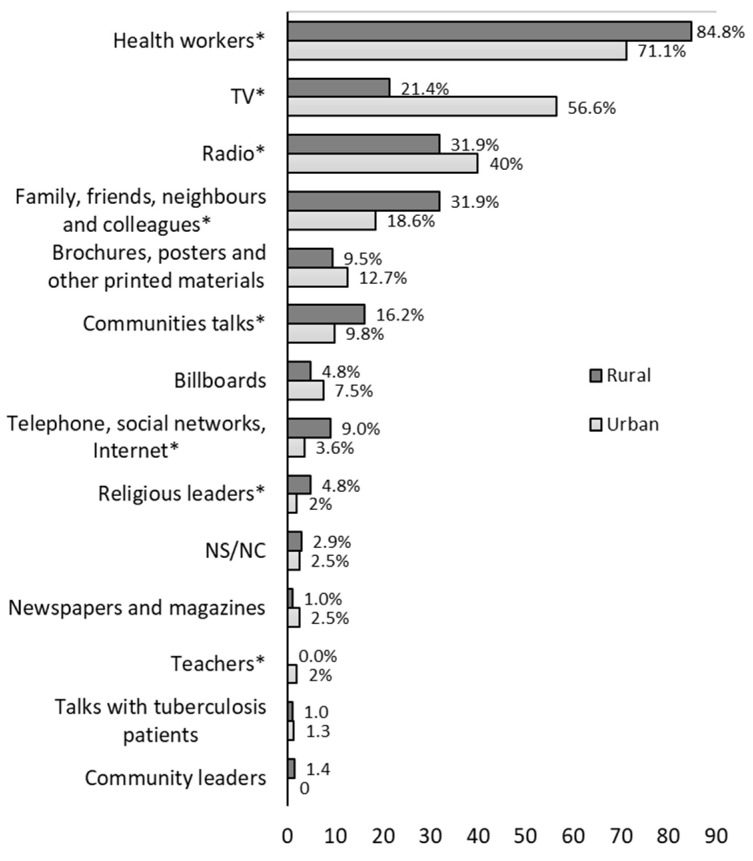
What are the sources of information that, in your opinion, can most effectively reach people like you with information about tuberculosis? * Significant chi-square test of differences by area at 5% level.

Figure 2 shows the sources of information where they hear talk about TB by area, the most frequently mentioned being health workers (62.4% rural vs. 47.1% urban), family and friends (60.0% vs. 47.1% *p* = 0.001), and TV (8.6% vs. 31.1% *p* < 0.001). Asked about which sources of information they considered could be the most effective to inform about TB (Figure 3), the most frequently mentioned source was health workers (84.8% rural vs. 71.1%, *p* < 0.001), followed by TV (21.4% vs. 56.6%, *p* < 0.001), the radio (31.9% vs. 40.0%, *p* = 0.039), and family, friends, neighbors, and colleagues (31.9% vs. 18.6%, *p* < 0.001).

### 3.1. Factors Associated to TB Knowledge

Caregivers aged 45 years or less had greater knowledge than those over 45 years old (PR: 1.30, 95% CI: 1.06–1.60) with no differences by sex. High knowledge about TB was also observed in those who have had TB (PR: 2.48, 95% CI: 1.87–3.30), or who had a family member with TB (PR: 1.49, 95% CI: 1.22–1.81). People with a higher level of education (PR: 1.37, 95% CI: 1.06–1.78) and richer wealth status (PR: 1.39, 95% CI: 1.01–1.92) had a higher level of knowledge about the disease’s transmission, prevention, and treatment. Table 2 shows the factors associated to high knowledge, good attitude, and low stigma towards TB.

### 3.2. Factors Associated to TB Attitudes

Differences by sex in TB attitudes were found, and women had a worse attitude than men (PR = 0.75, 95% CI: 0.62–0.91). Catholics are more likely to have better attitudes than caregivers from other religions (PR = 1.35, 95% CI: 1.04–1.76). Having a high TB knowledge score was associated with having a good attitude towards the disease (PR = 1.38, 95% CI: 1.15–1.67).

### 3.3. Factors Associated to TB Stigma

Respondents with wage employment were 1.21 times more likely to have a low TB-related stigma (95% CI: 1.02–1.44). Likewise, respondents who feel well informed (PR = 0.69, 95% CI: 0.58–0.83) and have a high TB knowledge score (PR = 1.34, 95% CI: 1.13–1.59) also had low TB-related stigma. Those who heard about TB from the radio (PR = 1.32, 95% CI: 1.05–1.65), billboards (PR = 1.86, 95% CI: 1.21–2.85), and family, friends, neighbors, or colleagues (P = 1.46, 95% CI: 1.20–1.77) had a lower TB stigma score than those who heard about TB from TV (95% CI: 0.59–0.98).

## 4. Discussion

This is the first study to assess KAP knowledge, attitudes, and stigma in Equatorial Guinea’s general population. Despite some positive attitudes towards the disease, most respondents had low awareness and knowledge about TB disease transmission, prevention and control, and a high TB-related stigma. Being younger than 45 years, having had a family member with TB, having a higher level of education, living in rural areas, and having a high socio-economic status were associated with higher knowledge of TB. Sex and religion were factors associated with attitudes towards TB, while work, information, and source of information were factors associated with TB-related stigma. The level of knowledge about the disease transmission and treatment was also a factor associated with both attitudes and stigma.

### 4.1. Knowledge

Knowledge about TB in Equatorial Guinea’s general population is low. Only one third of respondents had a high TB knowledge score, a percentage much lower than the knowledge found in other KAP studies in Africa [11,16,27,28,29]. Meanwhile, Guinean caregivers with a TB case in the family have better knowledge than the rest of the population. A good knowledge of the disease was also found in TB cases in a previous study in Equatorial Guinea [7]. This association could be due to the health education and counselling provided by health services when there is a TB patient in the household [11]. Education and communication activities should take into account the non-TB population in order to avoid unawareness in this group.

As in other studies, the knowledge of TB causes was low, and caregivers mentioned alcohol, tobacco, and drugs more frequently than bacteria or germs [30]. Furthermore, respondents mentioned that avoiding alcohol, tobacco and/or drugs was the best way to prevent TB. Although it is well known that alcohol and tobacco are risk factors for TB [31], there is a need to raise public awareness about the cause and prevention of TB to reduce transmission.

There is also a need to improve the Equatoguinean population’s knowledge of TB symptoms and transmission mechanisms. Only half of the population knew that TB is transmitted through the air when a person with TB coughs or sneezes and, although more than half of the respondents identified coughing as one of the main symptoms of TB, a high percentage did not know the symptoms of the disease. In addition, there is a widespread belief that TB is transmitted by sharing plates or glasses. TB is likely to spread more in the community if the population has low levels of knowledge about the causes, preventive methods, and transmission of the disease [30]. Misidentification of the signs and transmission of TB can also lead to community transmission and delays in seeking treatment [32].

People with secondary or higher education were 40% more likely to have good knowledge of TB than those with primary or lower education. This association has been found in other African countries [16,33,34,35]. A significant association between educational levels and knowledge was also observed in the study of TB patients in Equatorial Guinea [7], showing the relevance of tailoring messages to the less-educated general Equatorial Guinean population to improve not only TB awareness but also adherence to TB treatment [8].

### 4.2. Attitudes

Almost half of the respondents had a positive attitude toward tuberculosis in Equatorial Guinea, with women having a significantly worse attitude than men. Similar findings were found in The Gambia, where women also showed a worse attitude towards this disease [19]. Better attitudes in men could be explained by their higher incidence, because men accounted for 60% of TB cases in Equatorial Guinea in 2019 [4], implying that as TB patients, they may have better access to information about the disease. Having better attitudes towards TB is also associated with having good knowledge of the disease [19,30]. Usually, people who know the symptoms and how to prevent TB have better attitudes and healthcare seeking behavior, which reduces the delay in TB diagnosis and improves adherence to treatment [12].

This study also showed that Catholics in Equatorial Guinea have a better attitude compared to other religions. A similar result has been found in Lesotho, explained there by the solidarity described within the Catholic communities [36].

### 4.3. Stigma

More than half of the caregivers interviewed showed TB-related stigma. TB is a highly stigmatized disease, as it is usually associated with poverty, low socio-economic status, smoking, and alcohol consumption [37]. This stigma has been identified as a major barrier to patients seeking medical care and completing full treatment [38].

Beliefs such as that sharing household utensils can transmit TB cause patients to isolate themselves from their families and not participate in social activities [14]. In Equatorial Guinea, having wage employment and a high knowledge about the disease was found to be associated with low TB-related stigma [13,39]. Improving knowledge about disease transmission could overcome misconceptions and stereotypes and reduce stigmatization.

The use of mass media could be a double-edged sword, as some inappropriate media messages have been associated with TB stigma [40]. In Equatorial Guinea, respondents who reported receiving information about TB mainly through TV had higher TB-related stigma. In Ethiopia, patients and staff at a healthcare center explained that the television focuses information on how contagious TB can be and how severe the symptoms are, but gives little information on prevention, diagnosis, and treatment, leading to a lot of fear of being infected [41]. As in other studies [42], people who have received information about TB mainly through the radio and billboards have a lower level of stigma. In general, messages disseminated through radio and billboards are more tailored to the context and the local audiences [43].

Finally, people who receive information about TB from family, friends, neighbors, and colleagues had a lower level of stigma [39]. The community directly observed treatment strategy (C-DOTS) has shown how important it is to involve the community to improve TB health education, early diagnosis, treatment adherence, and stigma reduction [40].

The study has some limitations. First, it is a cross-sectional study, so the findings may not be generally applicable to very different contexts. Second, the WHO KAP TB questionnaire only includes some questions about stigma, so a specific study is needed to measure stigma.

## 5. Conclusions

A high percentage of the population in Equatorial Guinea is largely unaware of the causes, prevention, and transmission of TB, and this low knowledge of the disease is significantly associated with poor attitudes and high stigma, which often causes delay in diagnosis and low adherence to treatment. Given the epidemiological situation of TB in the country, there is an urgent need to improve knowledge and awareness of TB among the general population in Equatorial Guinea. The PNLP should update its health education strategy based on the identified gaps in caregivers’ knowledge and attitudes. These interventions should be contextualized to the local setting, designing appropriate messages to reach the most vulnerable populations, using appropriate information sources, and encouraging the participation of the community, people with TB, and health workers.

## Figures and Tables

**Table 1:** Socio-demographic characteristics of the sample by area.

Variable	Total (*n* = 770)	Rural (*n* = 210)	Urban (*n* = 560)	*p*-Value *
	*n* (%)	*n* (%)	*n* (%)	
** Sex **				0.008
Female	408 (53.0)	95 (45.2)	313 (55.9)	
Male	362 (47.0)	115 (54.8)	247 (44.1)	
** Age **				0.002
≤45 years	384 (49.9)	86 (41.0)	298 (53.2)	
>45 years	386 (50.1)	124 (59.0)	262 (46.8)	
** Marital status **				0.043
Widowed	103 (13.4)	37 (17.6)	66 (11.8)	
Married	413 (53.6)	116 (55.2)	297 (53.0)	
Single	243 (31.6)	53 (25.2)	190 (33.9)	
Divorced/separated	11 (1.4)	4 (1.9)	7 (1.3)	
** Educational status **				<0.001
Primary and lower	265 (34.4)	98 (46.7)	167 (29.8)	
Secondary	174 (22.6)	52 (24.8)	122 (21.8)	
Above secondary	331 (43.0)	60 (28.6)	271 (48.4)	
** Religion **				0.001
Catholic	616 (80.0)	185 (88.1)	431 (77.0)	
Other religions	154 (20.0)	25 (11.9)	129 (23.0)	
** Wage-employment **				<0.001
Yes	223 (29.0)	40 (19.0)	183 (32.7)	
No	547 (71.0)	170 (81.0)	377 (67.3)	
** Wealth **				<0.001
Poorest	154 (20.0)	110 (52.4)	44 (7.9)	
Second	153 (19.9)	67 (31.9)	86 (15.4)	
Middle	155 (20.1)	14 (6.7)	141 (25.2)	
Fourth	152 (19.7)	12 (5.7)	140 (25.0)	
Richest	156 (20.3)	7 (3.3)	149 (26.6)	
** Overcrowding index **				<0.001
≤1.4 points	385 (50.0)	140 (66.7)	245 (43.8)	
>1.4 points	385 (50.0)	70 (33.3)	315 (56.3)	
** Population type **				0.110
Non-TB cases	486 (63.1)	144 (68.6)	342 (61.1)	
TB case in the family	254 (33.0)	61 (29.0)	193 (34.5)	
TB case	30 (3.9)	5 (2.4)	25 (4.5)	

* Chi-square test.

**Table 2:** Factors associated to high knowledge, good attitude, and low stigma related to tuberculosis in Equatorial Guinea.

Variable (Reference)	High Knowledge	Good Attitude	Low Stigma
	PR (95% CI)	PR (95% CI)	PR (95% CI)
**Sex** (Male)			
Female		0.75 (0.62–0.91)	
**Age** (over 45 years old)			
45 years and less	1.30 (1.06–1.60)		
** TB case in the household (Non-TB cases) **			
TB case	2.48 (1.87–3.30)		
TB case in the family	1.49 (1.22–1.81)		
** Religion ** (Other religions)			
Catholic		1.35 (1.04–1.76)	
** Wage-employment ** (No)			
Yes			1.21 (1.02–1.44)
** Education level ** (Primary and lower)			
Secondary	1.20 (0.90–1.60)		
Above secondary	1.37 (1.06–1.78)		
** Wealth ** (Poor)			
Second to richest	1.39 (1.01–1.92)		
** Do you feel well informed about TB? ** (Yes)			
No			0.69 (0.58–0.83)
** Where have you heard about TB? ** (No)			
Radio—Yes			1.32 (1.05–1.65)
TV—Yes			0.76 (0.59–0.98)
Billboards—Yes			1.86 (1.21–2.85)
Family, friends, neighbors, colleagues—Yes			1.46 (1.20–1.77)
**TB knowledge Score (Low)**		1.38 (1.15–1.67)	1.34 (1.13–1.59)

Models were adjusted by sex and area (rural/urban). PR: prevalence ratio; 95% CI: confidence interval at 95% level.

## Data Availability

All data is included in the manuscript and supplementary material. The datasets analyzed during the current study are available from the corresponding author on reasonable request.

## Supplementary Materials

The following supporting information can be downloaded at: https://www.mdpi.com/article/10.3390/ijerph19148227/s1, Table S1: Tuberculosis symptoms and risk perception knowledge, Table S2: Tuberculosis transmission mechanisms and treatment knowledge and belief variables, Table S3: Attitudes towards tuberculosis disease, Table S4: Stigma towards patients with tuberculosis.
