# Supplementary material for: Knowledge, Attitudes, and Stigma: The Perceptions of Tuberculosis in Equatorial Guinea
Source: Int J Environ Res Public Health. 2022 Jul 6;19(14):8227. doi: 10.3390/ijerph19148227 (PMC9324553; doi:10.3390/ijerph19148227)
Supplement: Supplementary file 1 [file ijerph-19-08227-s001.zip › ijerph-1772018-supplementary.pdf]

Table S1. Tuberculosis symptoms and risk perception knowledge

	Total (n=770)		Rural (n=210)		Urban (n=560)		p-value*
	n	%	n	%	n	%	
**Do you think that there is TB in your community?**							<0.001
No	301	39.1	71	33.8	230	41.1	
Yes	250	32.5	100	47.6	150	26.8	
Do not know	219	28.4	39	18.6	180	32.1	
**Do you think TB is a serious disease?**							0.275
Yes	718	93.2	198	94.3	520	92.9	
No	32	4.2	6	2.9	26	4.6	
Do not know	19	2.5	5	2.4	14	2.5	
**What do you think causes TB?**							0.620
Do not know	484	64.6	139	66.5	345	63.9	
Alcohol, and tobacco and drugs	77	10.3	22	10.5	55	10.2	
Tobacco	59	7.9	13	6.2	46	8.5	
Bacteria or germs	57	7.6	12	5.7	45	8.3	
Alcohol	35	4.7	10	4.8	25	4.6	
Others	37	4.9	13	6.3	24	4.4	
**How is TB transmitted?**							
Sharing dishes/glasses	489	63.5	142	67.6	347	62.0	0.147
Through the air when a person with TB coughs or sneezes	389	50.5	99	47.1	290	51.8	0.251
Do not know	105	13.6	29	13.9	76	13.6	0.179
Through handshakes	56	7.3	8	3.8	48	8.6	0.023
Sexual relationship	30	3.9	9	4.3	21	3.8	0.732
By touching items in public places (doorknobs, handles in transportation, etc.)	26	3.4	6	2.9	20	3.6	0.625
**What are the signs and symptoms of TB?**							
Coughing	504	65.5	146	69.5	358	63.9	0.146
Weight loss	256	33.2	75	35.7	181	32.3	0.373
Do not know	162	21.0	40	19.0	122	21.8	0.406
Chest pain	121	15.7	28	13.3	93	16.6	0.266
Coughing up blood	116	15.1	40	19.0	76	13.6	0.058
Fever	102	13.2	26	12.4	76	13.6	0.664
Severe headache	25	3.2	6	2.9	19	3.4	0.709
Rash	6	0.8	1	0.5	5	0.9	0.558

* p-value of the Chi-square test of differences.

Table S2. Tuberculosis transmission mechanisms and treatment knowledge and belief variables

	Total (n=770)		Rural (n=210)		Urban (n=560)		p-value*
	n	%	n	%	n	%	
**In your opinion, who could be infected with TB?**							
Everybody	339	44.0	93	44.3	246	43.9	0.929
Only alcoholics	127	16.5	35	16.7	92	16.4	0.937
Do not know	92	11.9	29	13.8	63	11.3	0.329
Only drug users	85	11.0	21	10.0	64	11.4	0.573
Children	49	6.4	11	5.2	38	6.8	0.433
Smokers	37	4.8	16	7.6	21	3.8	0.025
Anyone who shares, is close to or lives with a TB patient (partner, family, etc.)	30	3.9	13	6.2	17	3.0	0.044
**Do you think tuberculosis can be prevented?**							0.314
Yes	604	78.4	157	74.8	447	79.8	
Do not know	104	13.5	33	15.7	71	12.7	
No	62	8.1	20	9.5	42	7.5	
**How?**							
Avoid sharing dishes	402	52.2	120	57.1	282	50.4	0.093
Covering mouth and nose when coughing or sneezing	234	30.4	56	26.7	178	31.8	0.169
Avoid shaking hands	71	9.2	13	6.2	58	10.4	0.075
Isolating the infected person	61	7.9	11	5.2	50	8.9	0.091
Hygiene	54	7.0	13	6.2	41	7.3	0.584
**Can TB be cured?**							0.227
Yes	754	97.9	205	97.6	549	98.0	
No	8	1.0	4	1.9	4	0.7	
Do not know	8	1.0	1	0.5	7	1.3	
**How?**							
With specific drugs	706	91.7	188	89.5	518	92.5	0.183
Praying	11	1.4	4	1.9	7	1.3	0.495
With herbal remedies	4	0.5	0	0.0	4	0.7	0.219
**Where do you think you should go to treat TB?**							0.222
Hospital	756	98.2	209	99.5	547	97.7	
Health Centre	11	1.4	1	0.5	10	1.8	
Do not know	3	0.4	0	0.0	3	0.5	
**How much do you think it costs to diagnose and treat tuberculosis?**							0.040
It is free of charge	571	74.2	167	79.5	404	72.1	
Do not know	124	16.1	25	11.9	99	17.7	
It is reasonably priced	38	4.9	8	3.8	30	5.4	
It is moderately expensive	16	2.1	2	1.0	14	2.5	
It is very expensive	17	2.2	8	3.8	9	1.6	
Other	4	0.5	0	0.0	4	0.7	

* p-value of the Chi-square test of differences.

Table S3. Attitudes towards tuberculosis disease.

Variable	Total (n=486) n (%)	Rural (n=144) n (%)	Urban (n=342) n (%)	p-value*
**Do you think you could get infected with TB?**				0.655
Yes	348 (71.6)	104 (72.2)	244 (71.3)	
No	94 (19.3)	25 (17.4)	69 (20.2)	
Do not know	44 (9.1)	15 (10.4)	29 (8.5)	
**How would you feel if you are diagnosed with TB?**				
Fear	167 (34.4)	52 (36.1)	115 (33.6)	0.598
Sadness or hopelessness	157 (32.3)	55 (38.2)	102 (29.8)	0.072
Rage	133 (27.4)	39 (27.1)	94 (27.5)	0.928
Surprise	64 (13.2)	20 (13.9)	44 (12.9)	0.761
I would cope with it	52 (10.7)	13 (9.0)	39 (11.4)	0.439
Shame	21 (4.3)	7 (4.9)	14 (4.1)	0.704
Do not know	20 (4.1)	6 (4.2)	14 (4.1)	0.970
**Who would you talk about your TB diagnoses?**				
Doctor or other medical worker	225 (46.3)	69 (47.9)	156 (45.6)	0.642
Other family member	210 (43.2)	80 (55.6)	130 (38.0)	<0.001
Partner	203 (41.8)	56 (38.9)	147 (43.0)	0.403
Parent	93 (19.1)	20 (13.9)	73 (21.3)	0.056
Child(ren)	43 (8.8)	12 (8.3)	31 (9.1)	0.796
Close friend	40 (8.2)	10 (6.9)	30 (8.8)	0.503
No one	4 (0.8)	2 (1.4)	2 (0.6)	0.370
Priest	3 (0.6)	1 (0.7)	2 (0.6)	0.888
Traditional healer	1 (0.2)	0 (0.0)	1 (0.3)	0.516
**Place to go if you would have TB symptoms**				
Hospital	452 (93.0)	136 (94.4)	316 (92.4)	0.419
Health care centre	36 (7.4)	9 (6.3)	27 (7.9)	0.527
Clinic/Private health care centre	6 (1.2)	0 (0.0)	6 (1.8)	0.110
Pharmacy	3 (0.6)	0 (0.0)	3 (0.9)	0.260
Church	3 (0.6)	1 (0.7)	2 (0.6)	0.888
Traditional healer	2 (0.4)	0 (0.0)	2 (0.6)	0.358
**If you do not improve, where would you go then?**				0.016
Hospital	341 (70.2)	114 (79.2)	227 (66.4)	
Traditional healer	44 (9.1)	12 (8.3)	32 (9.4)	
Church	18 (3.7)	1 (0.7)	17 (5.0)	
Health care centre	9 (1.9)	1 (0.7)	8 (2.3)	
Other	36 (7.4)	4 (2.8)	32 (9.4)	
Do not know	27 (5.6)	7 (4.9)	20 (5.8)	
Clinic/Private health care centre	8 (1.6)	3 (2.1)	5 (1.5)	
Pharmacy	3 (0.6)	2 (1.4)	1 (0.3)	

* p-value of the Chi-square test of differences.

Table S4. Stigma towards patients with tuberculosis

	Total (n=770)		Rural (n=210)		Urban (n=560)		p-value*
	n	%	n	%	n	%	
**Which statement is closest to your feeling towards people with TB?**							0.542
I feel compassion and desire to help.	572	74.3	149	71.0	423	75.5	
I feel compassion but I tend to stay away from these people	136	17.7	44	21.0	92	16.4	
I fear them because they can infect me	27	3.5	7	3.3	20	3.6	
I have no particular feeling	10	1.3	4	1.9	6	1.1	
It is their problem and I cannot get TB	4	0.5	1	0.5	3	0.5	
Other	10	1.3	1	0.5	9	1.6	
Do not know	11	1.4	4	1.9	7	1.3	
**In your community, how is a person who has TB usually treated?**							0.183
The community mostly supports and helps him or her	371	48.2	106	50.5	265	47.3	
Most people reject him or her	153	19.9	44	21.0	109	19.5	
Most people are friendly, but they generally try to avoid him or her	97	12.6	29	13.8	68	12.1	
They advise and help him or her	24	3.1	8	3.8	16	2.9	
Do not know	125	16.2	23	11.0	102	18.2	

* p-value of the Chi-square test of differences.
